# The success of vaginal birth by use of trans-labial ultrasound plus vaginal examination and vaginal examination only in pregnant women with labor induction: a comparative study

**DOI:** 10.1186/s12884-022-05324-4

**Published:** 2023-01-03

**Authors:** Elmira Nouri-Khasheh-Heiran, Ali Montazeri, Francesco Conversano, Maryam Kashanian, Mahboubeh Rasuli, Maryam Rahimi, Maryam Mirpour, Nahid Akbari

**Affiliations:** 1 Department of Reproductive Health and Midwifery, Iran University of Medical Sciences, Tehran, Iran; 2 Health Metrics Research Center, Iranian Institute for Health Sciences Research, The Academic Center for Education, Culture and Research (ACECR), Tehran, Iran; 3 Faculty of Humanity Sciences, University of Science and Culture, Tehran, Iran; 4 National Research Council, Institute of Clinical Physiology, Lecce, Italy; 5 Department of Obstetrics and Gynecology, School of Medicine, Iran University of Medical Sciences, Tehran, Iran; 6 Department of Biostatistics, Faculty of Health, Iran University of Medical Sciences, Tehran, Iran; 7 Department of Obstetrics and Gynecology, School of Medicine, Mashhad University of Medical Sciences, Mashhad, Iran

**Keywords:** Vaginal examination, Trans-labial ultrasound, Labor induction, Vaginal birth, Cesarean section, Angle of progression, Rotation angle

## Abstract

**Background:**

Predicting the success of vaginal delivery is an important issue in preventing adverse maternal and neonatal outcomes. Thus, this study aimed to compare the success rate of vaginal birth by using trans-labial ultrasound and vaginal examination, and vaginal examination only in pregnant women with labor induction.

**Methods:**

This was a comparative study including 392 eligible pregnant women with labor induction attending to a teaching hospital affiliated with Iran University of Medical Sciences from April to October 2018 in Tehran, Iran. Women were randomly assigned to two groups; the trans-labial ultrasound plus vaginal examination (group A), and the vaginal examination only (group B). Women were included in the study if they satisfied the following criteria: singleton pregnancy, 37 to 42 weeks of gestational age, fetal head presentation, a living fetus with no abnormalities, uncomplicated pregnancy, and no previous cesarean section or any uterine surgery. We used a partograph for both groups to assess the fetal head position and the fetal head station. In group 1, the Angle of Progression (AoP) and Rotation Angle (RA) were also assessed. Finally, the success and progression of vaginal delivery in two groups were compared by predicting the duration of delivery and mode of delivery.

**Results:**

The findings showed that 8.68% of women in the trans-labial plus vaginal examination group delivered by cesarean section, while 6.13% in the vaginal examination only group delivered by cesarean section (*P* = 0.55). In women with cesarean section in positive fetal head stations, Angle of Progression (AoP) was significantly decreased ranging from 90 to 135 degrees compared to women who delivered vaginally (135–180 degrees; *P* <  0.001). In addition, the Rotation Angle (RA) was significantly decreased in women with cesarean section ranging from 0 to 30 degrees compared to women who delivered vaginally (60-90degrees; *P* <  0.001). Further analysis indicated that a higher risk of cesarean section was associated with vaginal examination only as compared to trans-labial ultrasound plus vaginal examination (HR: 8.65, *P* <  0.001).

**Conclusion:**

Angle of Progression (AoP) and Rotation Angle (RA) indexes might be useful parameters to predict labor progression and successful vaginal delivery among women undergoing labor induction.

## Background

Globally cesarean section rates increased from around 7% in 1990 to 21% in 2018, and if this trend continues, by 2030 the highest rates are likely to be in Eastern Asia (63%), Latin America, Caribbean (54%), Western Asia (50%), Northern Africa (48%) Southern Europe (47%) and Australia and New Zealand (45%) [1]. However, the World Health Organization (WHO) has established the optimum cesarean section rate as 5 to 15% by medical indications [2–5].

In Iran, recent studies reported about seven-fold increase in the cesarean section rate; from less than 7% in the 1970s to over 48% in 2018 [4, 6–9]. The rate was reported to be higher in private hospitals (72–89%) [10–13].

Cesarean section such as any other surgeries could lead to a variety of complications and was commonly performed for mothers with history of previous cesarean section, fetal distress, and prolonged labor [14]. To prevent prolonged labor and ending pregnancy with vaginal birth, some procedures such as induction with oxytocin and prostaglandins via monitoring the labor process were used [15].

Labor monitoring and predicting the likelihood of vaginal birth following induction has become important in this era of personalized medicine, not just from women’s perspectives, but also to ensure optimal allocation of healthcare resources [16]. Particularly, labor is usually monitored to ensure that there are no signs of abnormal progress that might be harmful to mother or baby. The method most commonly used is routine vaginal examination (undertaken at regular time intervals) in order to provide information on cervical dilatation and the position of the baby [17]. However, evidence suggests that the manual vaginal examination might lead to errors (up to 88% of cases) and has limitations in indicating labor progression [18–23]. As such, management of childbirth needs new approaches and guidelines, exploiting objective indications for standardized quantitative monitoring, and appropriate medical decision making for early and correct identification of the most effective mode of delivery [18, 24, 25].

Recent studies demonstrated that ultrasound techniques are very helpful in the measurement of labor progression parameters [20, 25–30]. For instance, the evaluation of the Progression Angle (PA) or Angle of Progression (AoP), and Rotation Angle (RA) by trans-labial ultrasound imaging provides an objective, accurate, and reproducible method for determining fetal head progression during labor [27, 31]. Since in Iran using trans-labial ultrasound is not common in hospitals for monitoring labor procedures, this study aimed to evaluate the success rate of vaginal birth using trans-labial ultrasound plus vaginal examination compared to vaginal examination only in pregnant women with labor induction.

## Methods

### Design and participants

This was a comparative study. The study was conducted from April to October 2018. The study participants consisted of a cohort of pregnant women candidates for labor induction attending to the maternity ward in a teaching hospital affiliated with the Iran University of Medical Sciences in Tehran, Iran. Candidates were selected based on the inclusion criteria. The inclusion criteria were: to be Iranian, singleton pregnancy, 37 to 42 weeks gestational age (with ultrasound history in the first or second trimester of pregnancy), fetal head presentation, a living fetus with no abnormalities (known for embryos along with ultrasound reports), uncomplicated pregnancy (including preterm labor, placental abruption, severe preeclampsia, marginal placenta previa), no history of previous cesarean section or any uterine surgery, estimated fetal weight less than 5 kg, and not being estimated maximum one station of the fetal head during labor (with regard to the unpredictable speed prenatal delivery in women specially in the precipitous labor). The exclusion criteria were: cephalo-pelvic disproportion, and unwillingness to participate in the study. We used two methods to collect the data: ‘trans-labial ultrasound plus vaginal examination’, and ‘vaginal examination only’. Thus, women were randomly assigned into two groups: trans-labial ultrasound plus vaginal examination (group A) and vaginal examination only (group B). As such a standard-sized paper marked A (trans-labial plus vaginal examination group) or B (vaginal examination only group), and folded to fit an envelope. Then, at the time of admission to the delivery room, one of us (ENKH), offered a blind envelop to women for group assignment.

### Sample size

In order to detect 20% differences in success rate between group A and group B (*P*_1_ = 90% success rate for group A and 70% success rate for group B) we used the following formula to estimate sample size.

n = (Z_α/2_ + Z_β_)^2^ * [p_1_(1-p_1_) + p_2_(1-p_2_)]/(p_1_-p_2_)^2^.

Considering a power of 80% and type I error equal to 5%, a sample size of 196 women per each group was estimated.

### Trans-labial ultrasound

**Fig. 1:**
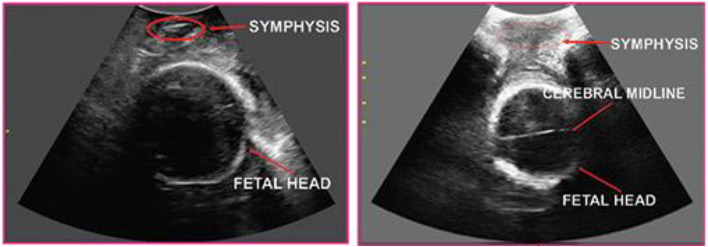
Eco view panel of Anatomical points in Ultrasound’s measurement

**Fig. 2:**
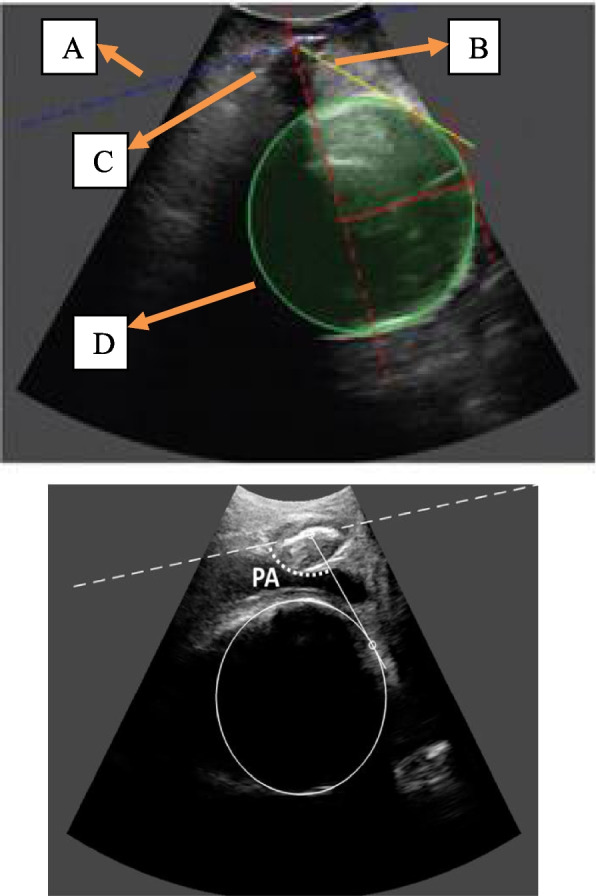
Eco view panel of measuring AoP

**Fig. 3:**
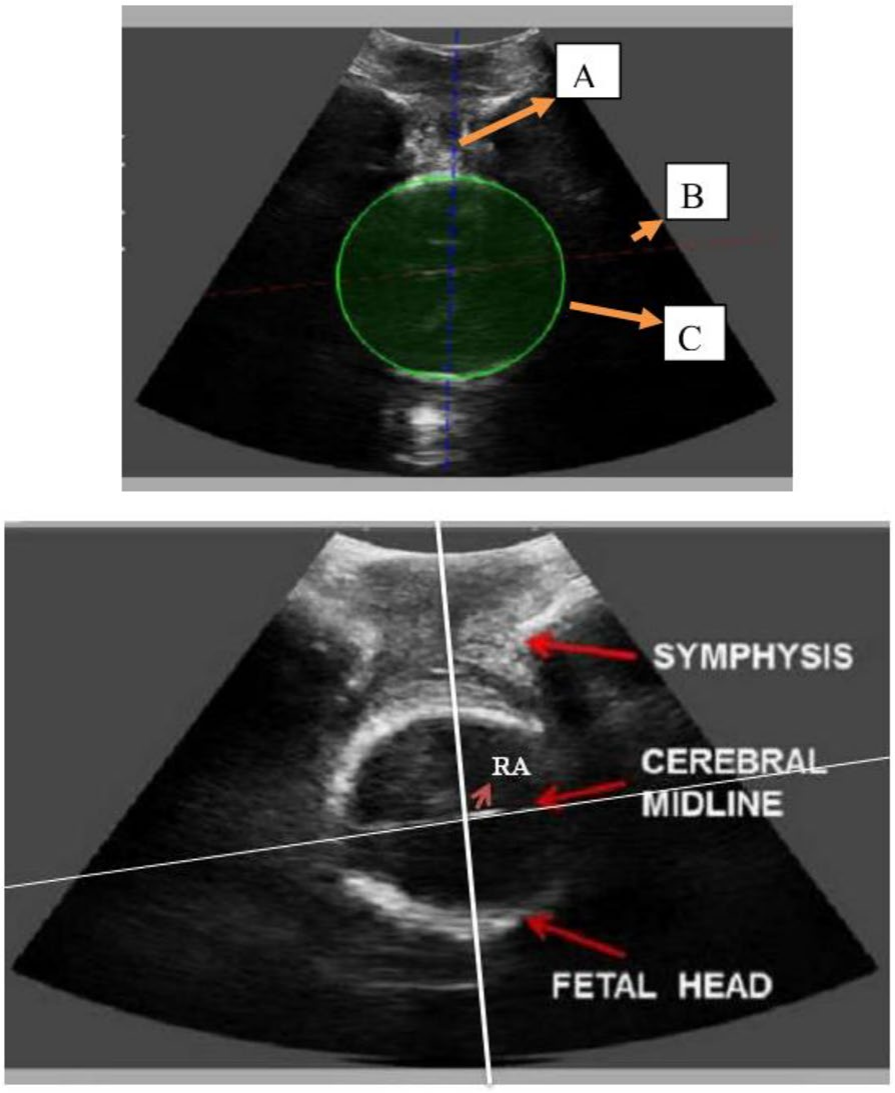
Eco view panel of measuring RA

**Fig. 4:**
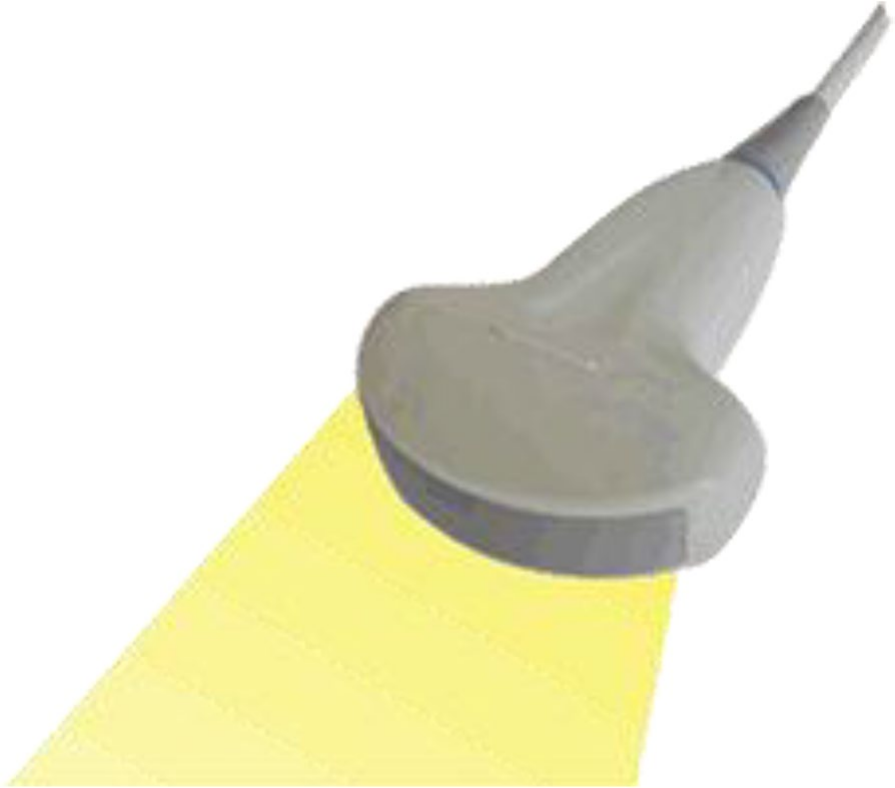
Ultrasound probe

**Fig. 5:**
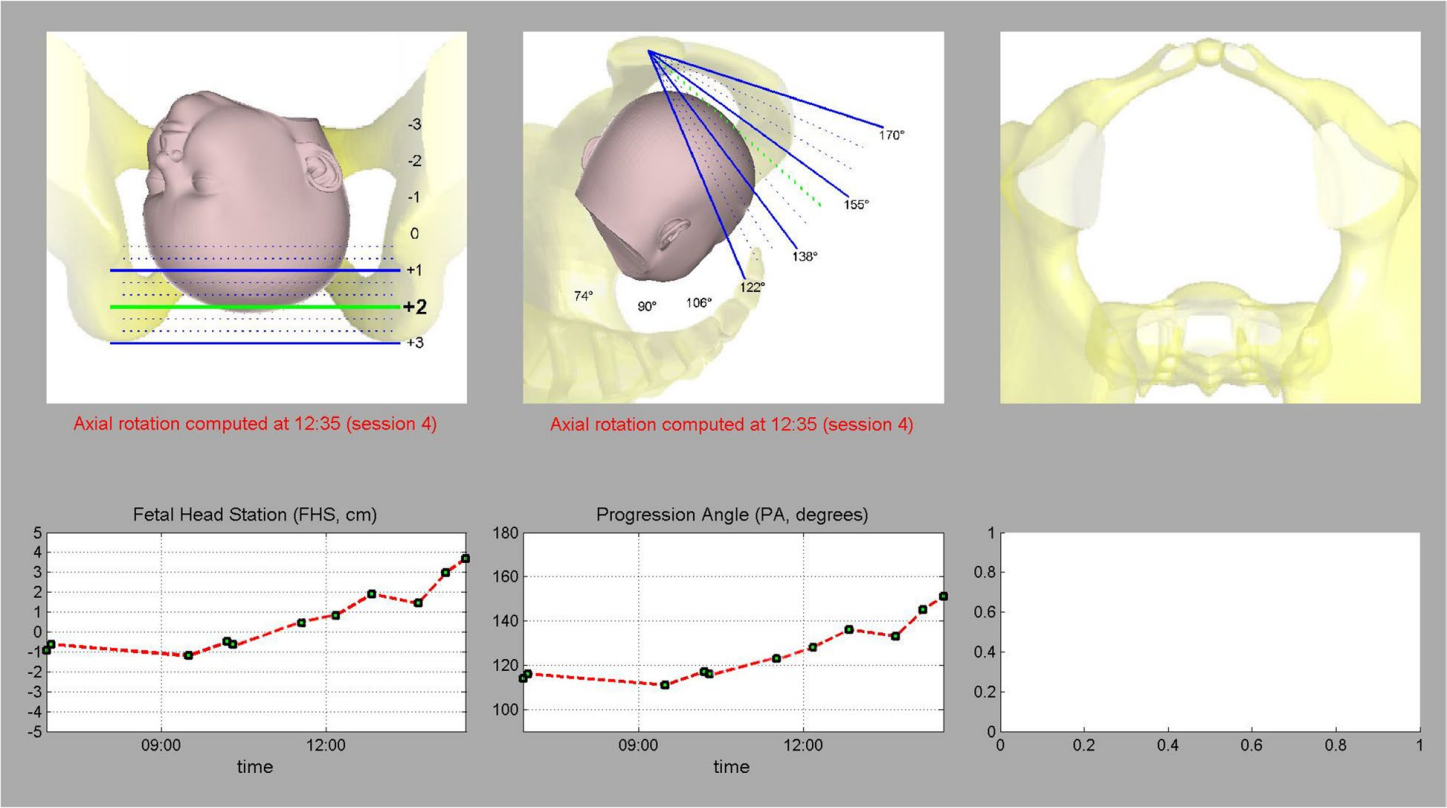
3D show of station and AoP of fetal head

**Fig. 6:**
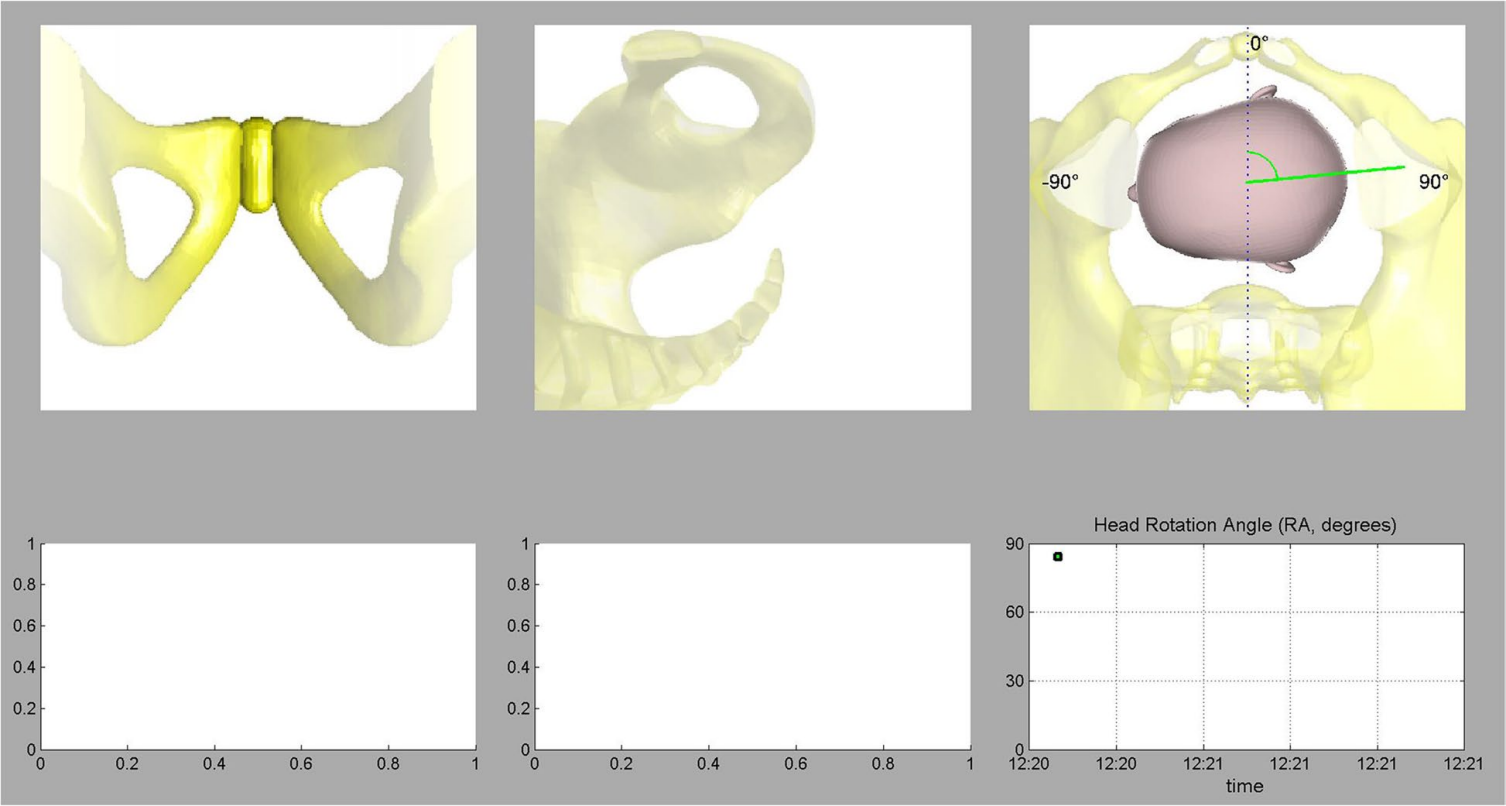
3D show of RA

**Fig. 7:**
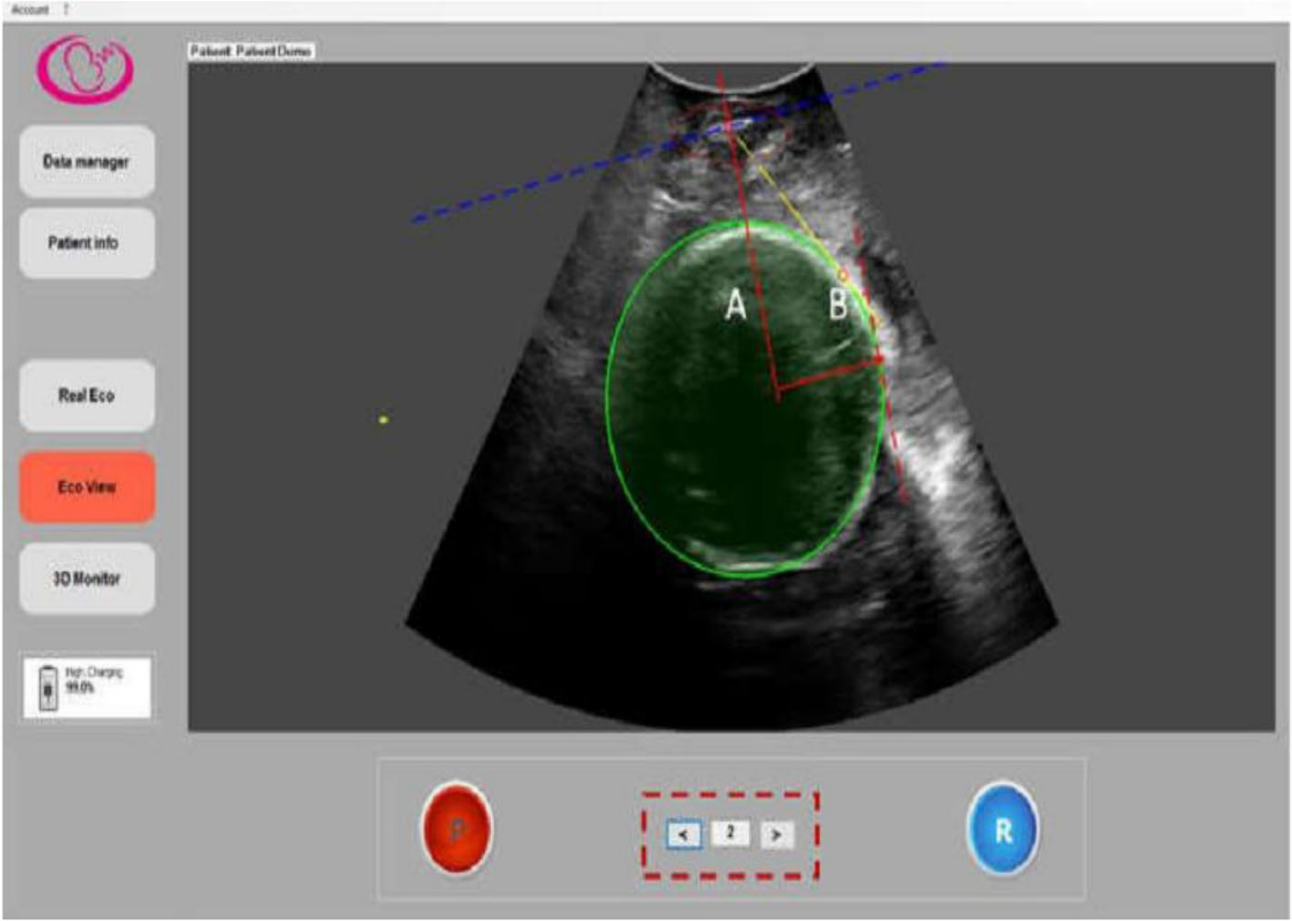
Eco view panel

- *Procedure:* Pregnant women were placed in a lithotomic position while their bladder was empty. The main investigator (ENKH) placed a trans-labial ultrasound probe in the trans-labial space of women. All underwent trans-labial ultrasound were performed by the main investigator. The ultrasound scan was equipped with a real-time tracking algorithm designed to guide the operator through the acquisition, with an automatic identification of anatomic landmarks (Fig. 1), in order to be able to measure fetal head station, fetal head position, Angle of Progression and, Rotation Angle during labor (Fig. 2 and Fig. 3). The probe was the Canox probe 3.5 MHz. It allowed quantitative monitoring of labor instantly through an automatic description of ultrasound images (Fig. 4). Afterwards, the probe was enclosed in a latex glove covered with ultrasound gel and then placed between the labia, below the pubic symphysis. In each occasion ultrasound imaging (the first probe) was placed vertically and data of the Angle of Progression and fetal head station were recorded (Fig. 5). Consequently, the probe was rotated 90 degrees to the right hip of the women, and data from the fetal head position and Rotation Angle were recorded (Fig. 6). Repeated imaging was performed by recording the time of imaging and a partograph was recorded in order to identify the labor process including information on the fetal head station and position, Angle of Progression, and Rotation Angle (Fig. 5 and Fig. 6). The trans-labial ultrasound was performed every 4 hours in the first stage of labor, and every 1-hour or more in the active phase of labor based on the number of vaginal examinations.
- *Measurement:* Trans-labial ultrasound data recording was performed for 5 seconds immediately after each uterine contraction using trans-labial ultrasound. We used a trans-labial ultrasound that automatically determines anatomical landmarks of bone structure and measures the most important indicators of progress: fetal head station, fetal head position, Angle of Progression or Progression Angle (AoP or PA), and Rotation Angle (RA) (Fig. 2 and Fig. 3). The AoP (or PA) is the angle between the longitudinal axis of the symphysis pubis (blue dotted line in Fig. 2) and the tangent line to the fetal head (yellow dotted line in Fig. 2). In fact, the AOP is a measure between a line placed through the midline of the pubic symphysis and a line running from the inferior apex of the symphysis pubis tangentially to the fetal skull (Fig. 2 and Fig. 7). The machine also automatically calculates the fetal head station value of the images obtained. To calculate the RA, the reference parameters (anterior-posterior axis of the mother’s pelvis (blue dotted line in Fig. 3), the surrounding area of the fetal head (green circle in Fig. 3), and the midline of the fetal head (red dotted line in Fig. 3) were used. Then, three-dimensional reconstructions with a graphical and temporal representation of the measured parameters were provided (Fig. 5 and Fig. 6).
- *Normal progress in trans-labial ultrasound*: The partograph has been heralded as one of the most important advances in modern obstetric care. The World Health Organization (WHO) advocates its use as a necessary tool in the management of labor. In the normal active phase of labor, plotting of the fetal head station will have a four-hour progress. If it lasts more than four-hour, labor may be prolonged [32].
- *Abnormal progress in trans-labial ultrasound*: Abnormal labor included protracted and arrest disorders. Protraction disorders refer to protracted active phase descent. Descent 1 cm/h in primiparas and 2 cm/h in multiparas is protracted descent. Arrest disorders refer to the secondary arrest of the fetal head descent for more than 1 hour [33].

### Vaginal examination

- *Procedure:* Pregnant women were placed in a lithotomic position whilst their bladder was empty. In women who were able to pass urine, especially in the latent phase of labor, it was encouraged to do it before every manual vaginal examination or trans-labial ultrasound, and in those who had received anesthesia methods or were in the active phase of labor and were unable to do it physiological, urinary catheterization was used. Vaginal examination was performed by a midwife. The midwife was blinded to the study, and confirmed by three senior gynecologists (there was no extra touch. The gynecologists just were observers in order to see if the midwife performs vaginal examination correctly). The findings of the examination, including information about fetal head station, fetal head position and labor process were recorded in the delivery form and partograph. Vaginal examination was performed every 4 hours in the first stage of labor, and every 2-hour or more in the active phase of labor [15]. Vaginal examination data was carried out after each uterine contraction.
- *Measurement:* Partograph was drawn with the most important indicators of progress: the fetal head position and the fetal head station. Since we did not need other information such as cervical dilatation for this study, thus we did not record extra information. The rate of progression of the labor is presented in Fig. 5.

### Outcome measure

The primary outcome of this study was the success in predicting vaginal delivery by either group. As such predicting delivery time, and mode of delivery were considered.

### Statistical analysis

The findings were analyzed using descriptive statistics (mean, standard deviation, and frequency distribution). To compare data between the two methods (trans-labial ultrasound plus vaginal examination and vaginal examination only) independent *t*-test, analysis of variance, and Chi-square as well as Mann-Whitney U test, and Kruskal-Wallis test were performed as appropriate. Sensitivity analysis was used in order to compare the two methods in predicting vaginal delivery time. Additionally, the Cox proportional hazards regression model was used to estimate the probability of delivery outcomes while controlling for independent variables. Independent variables included a number of demographic information (date of birth, mother’s education level, mother’s occupation, gestational age, weight gained during pregnancy, height, current weight, pregnancy stage, prescription of pain medication or non-receipt of pain medication and Bishop’s score.) and risk factors (prolong labor, fetal distress due to abnormal fetal heart rate or umbilical cord prolapse and placental separation). Finally, survival analysis was performed to predict delivery time. Then we compared the prediction time between the two study groups by t-test. All statistical analysis was performed using the SPSS 16 software.

## Results

### Participants

A total of 392 pregnant women were included in the study and they were randomly assigned into two groups. The mean age of participants in the ‘trans-labial plus vaginal examination’ group was 27.59 (SD = 6.24) years, and it was 27.49 (SD =6.44) years for the ‘vaginal examination only’ group. There were no significant differences between the two groups in terms of age. Also, there were no significant differences in mothers’ education (*P* = 0.4), gestational age (*P* = 0.8), and the number of pregnancies (*P* = 0.2) between the two groups (Table 1).

**Table 1:** Baseline characteristics of the study sample*

	Vaginal examination only (***n*** = 196)	Trans-labial ultrasound plus vaginal examination (***n*** = 196)	***P***-value
Mother’s age (mean, SD)**	27.49 (6.44)	27.59 (6.24)	0.78
**Mother’s education** ***			0.40
None	27 (13.77)	19 (9.69)	
Primary	40 (20.41)	39 (19.89)	
Secondary	118 (60.21)	122 (62.25)	
Higher	11 (5.61)	16 (8.17)	
Gestational age for termination of pregnancy in week (mean, range)**	39 (38–40)	39 (38–40)	0.87
Body Mass Index (BMI) (mean, SD)**	29.81 (4.25)	29.87 (4.65)	0.944
Estimated fetal weight in gram (mean, SD)**	3100(383.5)	3200 (385.0)	0.016
Anesthesia method***			0.002
Spinal Analgesia	59(30.10)	104(53.06)	
Epidural analgesia	6(3.06)	3(1.53)	
Pain relievers by intramuscular injection	109(55.61)	62(31.63)	
Without any pain relieving methods	22(11.23)	27(13.78)	
**Indication of induction** ***			0.87
High blood pressure without preeclampsia	5(2.55)	3(1.53)	
Decreased intrauterine growth / oligohydramnios	5(2.55)	3(1.53)	
Diabetes mellitus	4(2.03)	5(2.55)	
Duration of delivery	57(29.80)	62(31.63)	
Rupture of the amniotic sac	80(40.80)	76(38.78)	
Others	45(22.90)	47(23.98)	
**Mode of induction** ***			0.501
Oxytocin	184(93.88)	187(95.41)	
Misoprostol	12(6.12)	9(4.59)	
Indication for cesarean section***			0.28
Prolong Labor	2(16.66)	4(23.52)	
Fetal distress with meconium in the process of induction of labor	2(16.66)	1(5.88)	
Fetal distress caused by an abnormal fetal heart pattern in the process of induction of labor	6(50.00)	12(70.58)	
Others	2(16.66)	0 (0.00)	
**Number of Pregnancies** ***			0.21
Nulliparous	87(44.38)	83(42.34)	
Multiparous	109(55.62)	113(57.66)	
Final position of fetal head ***			0.30
Anterior occiput	193(98.47)	190(96.94)	
Posterior occiput	3(1.53)	6(3.06)	
Type of delivery ***			0.51
Vaginal delivery	184(93.87)	179(91.32)	
Cesarean delivery	12(6.13)	17(8.68)	

* All values are frequency and (percentage) except for those indicated as mean and standard deviation

**Derived from t-test

***Derived from Chi-squared

### Risk of cesarean section

The results obtained from Cox’s proportional hazard for the probability of the outcome measure (type of delivery) among the two study groups are shown in Table 2. As indicated there was a higher risk of cesarean section associated with vaginal examination only as compared to ultrasound labial plus vaginal examination (HR: 8.65, *P* <  0.001).

**Table 2:** Cox’s proportional hazards regression model for estimating risk of cesarean section*

	Regression Coefficient	SD	Wald Statistic	Hazard Ratio (HR)	***P***-value
Group					
Trans-labial ultrasound plus vaginal examination	–	–	–	1.0 (ref.)	–
Vaginal examination only	2.16	0.598	13.01	8.65	< 0.001
Age	0.061	0.035	2.954	1.065	0.086
Mother’s current weight	0.031	0.053	0.330	0.970	0.566
BMI	0.075	0.143	0.271	1.077	0.603
Gestational age	0.078	0.279	0.078	0.925	0.779
Frequency of pregnancy	0.676	0.269	6.327	0.509	0.012
Weight at birth	0.002	0.001	5.846	0.998	0.016
The final position of the fetal head					
OA	–	–	–	1.0 (ref.)	–
OP	1.39	0.696	4.04	4.053	0.034

* This analysis predicts that women who received vaginal examination only were more than 8.65 times less likely (less successful) to proceed with vaginal delivery compared to the trans-labial ultrasound and vaginal examination group

### Prediction of duration of delivery mode

Survival analysis was used to predict the duration of delivery (in minutes) for the two groups. The duration was estimated from the beginning of induction to the time of delivery (whether vaginal or cesarean section). The results showed that the prediction of duration of vaginal delivery was 144.88 ± 99.25 minutes in the trans-labial plus the vaginal examination group and 432.99 ± 267.71 in the vaginal examination only group (P<0.01, Table 3). The time-course analysis showed that the prediction of duration for vaginal delivery in the ultrasound method was lower than in the manual vaginal examination method at all instances.

**Table 3:** Prediction of the duration of vaginal delivery (minutes) in the two study groups

	Trans-labial ultrasound plus vaginal examination group (***n*** = 196)	Vaginal examination only group (***n*** = 196)
Mean ± SD	144.88 ± 99.25	432.99 ± 267.71
Range	15–480	25–1020
*P*-value (Mann-Whitney U test)	< 0.001	

### Sensitivity analysis

The findings showed that the sensitivity for prediction of vaginal birth with manual vaginal examination was 75.54% and by trans-labial ultrasound it was 91.6%. The results also showed that the data obtained from trans-labial ultrasound were more sensitive in predicting vaginal birth, especially in zero and positive stations (Tables 4 and Table 5).

**Table 4:** Frequency distribution and partograph sensitivity of fetal head descending progression in trans-labial ultrasound plus vaginal examination group

	Vaginal delivery		Cesarean section	
	No.	%	No.	%
Normal progress	164	91.6	7	41.1
Abnormal progress	15	8.4	10	58.9
Total	179	100	17	100
Chi-square test results (Fisher’s exact distribution)	*χ*^2^=23.51, df = 1, *P* < 0.001			
Sensitivity of trans-labial ultrasound plus vaginal examination	91.6%

**Table 5:** Frequency distribution and partograph sensitivity of fetal head descending progression in vaginal examination only group

	Vaginal delivery		Cesarean section	
	No.	%	No.	%
Normal progress	139	75.5	3	25.0
Abnormal progress	45	24.5	9	75.0
Total	184	100	12	100
Chi-square test results (Fisher’s exact distribution)	*χ*^2^=14.418, df = 1, *P* < 0.0001			
Sensitivity of vaginal examination only	75.54%			

### Progression angle and rotation angle

**Fig. 8:**
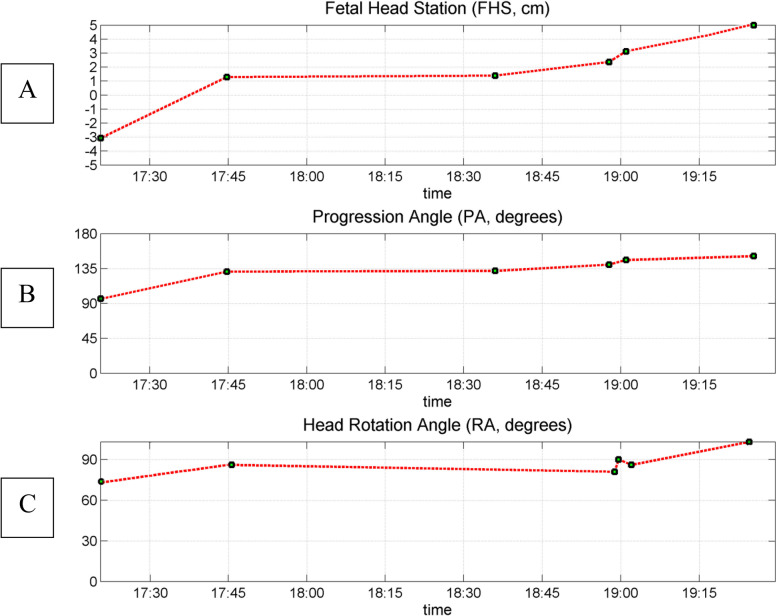
Eco view panel of final charts leading vaginal delivery

**Fig. 9:**
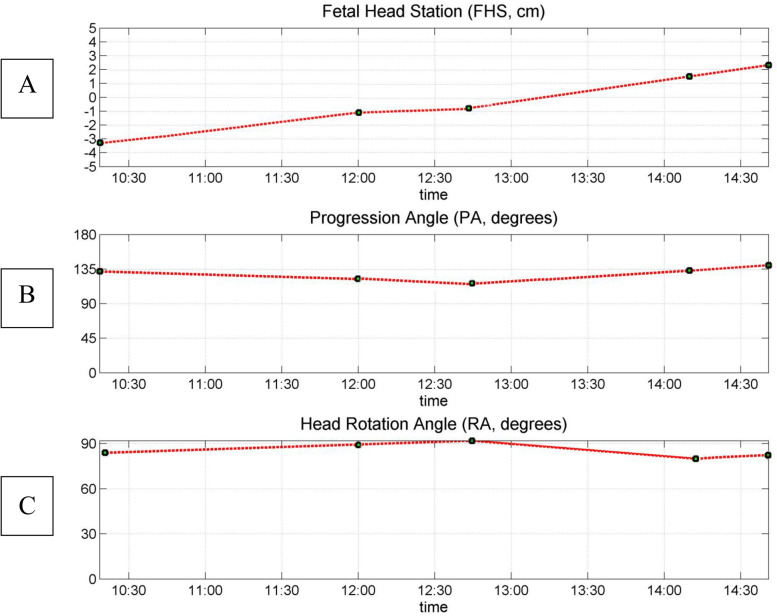
Eco view panel of final charts leading cesarean section

Finally, when the progression angle was measured in the trans-labial ultrasound group the findings indicated that for women whose progression angle in 0 and + 1 station was 135 degrees, vaginal birth occurred (Fig. 8), while for women whose progression angle in 0 and + 1 stations was less than 135 degrees, cesarean section occurred (Fig. 9). Also, in stations + 2 and + 3, vaginal birth occurred for those women who had progression angle of 135 to180 degrees (Table 6). In addition, while the majority of women in the vaginal birth group (57.5%) had the final rotation angle of the fetal head within 60 to 90 degrees, the majority of women in the cesarean group (47.1%) had the final angle of 0 to 30 degrees (Table 7).

**Table 6:** Comparison between head angle to the pubic bone center (angle progression) and sensitivity to the segregation of the fetal head and the prediction of the type of final delivery (cesarean/vaginal) in the two groups

Station	Sensitivity	Vaginal delivery		Cesarean section		***P***-value
		No.	AoP	No.	AoP	
0	98	102	135^°^	6	90–135^°^	0.135
+ 1	97.4	108	135^°^	8	90–135^°^	0.046
+ 2	93.6	136	135–180^°^	6	135^°^	0.007
+ 3	91.2	170	135–180^°^	6	135^°^	< 0.001

**Table 7:** End-to-end rotation of the fetal head in the trans-labial ultrasound group (196) in women with singleton and preterm labor and its relation to the type of vaginal delivery or cesarean section

	Vaginal delivery		Caesarian section	
	No.	%	No.	%
No sampling	4	2.2	1	5.9
30° to 0°	29	19.6	11	64.7
30°	7	3.9	1	5.9
60° to 30°	19	10.6	1	5.9
60°	11	6.2	1	5.9
90° to 60°	109	57.5	2	11.7
Total	179	100	17	100
Chi-square test results	*χ*^2^=22.71, df = 7, *P* < 0.001			

## Discussion

The findings from this study indicated that trans-labial ultrasound was more effective than manual vaginal examination in the labor process, and accurate prediction of delivery types. Similar findings were reported from studies that assessed AoP and RA. They showed that trans-perineal ultrasound could successfully predict vacuum extraction failure and vaginal birth following induction of labor [34–39].

The findings indicated that the prediction of duration of vaginal birth was increased if the Angle of Progression in zero and positive stations locate at 135 to 180 degrees (Table 6). However, by separating the fetal head station, we introduced some intervals for the Angle of Progression that would have increase the probability of vaginal birth with 91.6% sensitivity (Table 4). A recent study showed that if fetal head angle locates between 135 and 158 degrees in the second phase of labor, the probability of success significantly would increase for instrumental birth. The same study reported that when Angle of Progression was less than 135 degrees, the success of vaginal birth (with or without instrument) decreased [36].

The current study showed that sensitivity for trans-labial ultrasound was 91.6%, while sensitivity for the routine vaginal examination was 75.54% (Table 4, and Table 5). Such findings suggest that trans-labial ultrasound was more accurate in predicting vaginal birth. Therefore, to assess labor progress it is recommended to use trans-labial ultrasound as an adjunct to routine vaginal or digital clinical examinations to reduce invasive procedures and human errors. Similarly, studies have shown that ultrasound, as a less invasive and reproducible method, was effective in predicting the success of vaginal birth in pregnant women compared to other methods [22, 40, 41]. As such studies reported that the accuracy of the ultrasound method did not significantly change, even if the examiners had different levels of proficiency [18, 28, 42, 43] while this is not true for vaginal examination [44].

Although the present study was focused on assessing the prediction of duration of vaginal delivery and the AoP measurement by expert manual segmentation, we also found a good agreement between a range of AoP values and the occurrence of a spontaneous delivery. In particular, in the present study, for all women who delivered vaginally the value for AoP was greater than 135 degrees in the zero station, and 135 to 180 degrees in positive stations of the fetal head (Table 6). A recent study stated that the late diagnosis of posterior occiput position in the second stage of labor and obstructed labor is exposed to a high risk of maternal and fetal morbidity [45]. Moreover, other studies reported that using ultrasound technique and the earlier detection of the abnormal fetal head station is more precise than vaginal examination which might lead to maternal complications and maternal and neonatal unfavorable outcomes [21, 37, 46].

The findings from the current study showed that the majority of women with a final RA of the fetal head above 60 degrees had vaginal birth while the value for those who had cesarean section was less than 30 degrees (Table 7). However, a study reported that women with a final RA of the fetal head above 22 degrees experienced vaginal birth [41]. Another study stated that women with a final RA of the fetal head above 45 degrees experienced cesarean deliveries, and those with less than 45 degrees experienced vaginal birth [26]. Such differences might be due to differences in observation points in trans-labial ultrasound. Technicians might see the central point of the pubic bone while experienced sonographers usually measure the peripheral of the pubic bone. The other possibility for such observation was the fact that in the current study the data obtained from the processing of echo graphic images in the trans-labial ultrasound, automatically created the progress algorithms without human manipulation which significantly decreased human error.

## Strengths and limitations

In order to measure AoP we used the center of pubis symphysis bone while previous studies used peripheral sides for such measure [24, 36]. Using peripheral sides firstly needs a highly experienced expert and secondly might introduce more errors. In addition, the current study benefited from a relatively large sample size compared to previous investigations on the topic. However, the study had some limitations. Since this study was an observational study, there was a difference between women in the two study groups with regard to the number of pregnancies that might influence the findings. Studies with more homogenous samples are recommended.

## Conclusion

Overall, the findings suggest that trans-labial ultrasound plays a significant role in the management of labor and helps to make the right decisions in the labor process. As such the Angle of progression (AoP) and Rotation Angle (RA) measured by trans-labial ultrasound were influential factors to predict vaginal birth or cesarean section in labor process. Indeed, further investigations on the use of trans-labial ultrasound for the prediction of successful vaginal birth are recommended.

## Data Availability

The data are available from corresponding authors on reasonable request.

## Abbreviations

Aop/ PA: Angle of Progression/ Progression Angle
OA: Occiput anterior
OP: Occiput posterior
RA: Rotation Angle
IUGR: Intra uterine growth reduction
GDM: Gestational diabetic mellitus
ROM: Rupture of the amniotic sac

## References

[1] Betran AP, Ye J, Moller AB, Souza JP, Zhang J (2021). Trends and projections of caesarean section rates: global and regional estimates. BMJ Glob Health.

[2] Daemi A, Ravaghi H, Jafari M (2019). Risk factors of neonatal mortality in Iran: a systematic review. Med J Islam Repub Iran.

[3] Harrison MS, Goldenberg RL (2016). Cesarean section in sub-Saharan Africa. Matern Health Neonatol Perinatol.

[4] Betrán AP, Torloni MR, Zhang JJ, Gülmezoglu A, Aleem H, Section WWGoC (2016). WHO statement on caesarean section rates. BJOG.

[5] World Health Organization (1985). Appropriate technology for birth. Lancet.

[6] Ahmad Nia S, Delavar B, Eini Zinab H, Kazemipour S, Mehryar A, Naghavi M (2009). Caesarean section in the Islamic Republic of Iran: prevalence and some socio-demographic correlates. EMHJ.

[7] Badakhsh MH, Seifoddin M (2012). Rise in cesarean section rate over a 30-year period in a public hospital in Tehran. Arch Iran Med.

[8] Rafei M, Ghare MS, Akbari M, Kiani F, Sayehmiri F, Sayehmiri K (2018). Prevalence, causes, and complications of cesarean delivery in Iran: a systematic review and meta-analysis. Int J Reprod Biomed.

[9] Abedian Z, Navaee M, Jaafari Sani H, Arani A, Ebrahimzadeh S (2012). Comparing the effect of two teaching methods, role playing and lecture on primigravida women’s knowledge, attitude and performance according to delivery mode. Iran J Obstet Gynecol Infertil.

[10] Yavangi M, Sohrabi M-R, Alishahi TA (2013). Effect of Iranian ministry of health protocols on cesarean section rate: a quasi-experimental study. J Res Health Sci.

[11] Azami-Aghdash S, Ghojazadeh M, Dehdilani N, Mohammadi M (2014). Prevalence and causes of cesarean section in Iran: systematic review and meta-analysis. Iran J Public Health.

[12] Omani-Samani R, Mohammadi M, Almasi-Hashiani A, Maroufzadeh S (2017). Cesarean section and socioeconomic status in Tehran. Iran J Health Sci.

[13] Mohamadbeigi A, Tabatabaee SH, Mohammad Salehi N, Yazdani M (2009). Factors Infuencing cesarean delivery method in shiraz hospitals. Iran J Nurs Res.

[14] Dorji T, Wangmo K, Dorjey Y, Dorji N, Kiran Chhetri D, Tshering S, Wangmo P, Tshokey T (2021). Indications and factors associated with cesarean section in Bhutan: A hospital-based study. Int J Gynecol Obstet.

[15] Canningham F, Leveno KE, Bloom ST, Hauth JO, Rouse DW, Spong CA (2010). Williams obstetrics.

[16] Alavifard S, Meier K, Shulman Y, Tomlinson G, D'Souza R (2019). Derivation and validation of a model predicting the likelihood of vaginal birth following labour induction. BMC Pregnancy Childbirth.

[17] Moncrieff G, GML G, Dahlen HG, Thomson G, Singata-Madliki M, Clegg A, Downe S (2022). Routine vaginal examinations compared to other methods for assessing progress of labour to improve outcomes for women and babies at term. Cochrane Database Syst Rev.

[18] Conversano F, Peccarisi M, Pisani P, Di Paola M, De Marco T, Franchini R (2017). Automatic ultrasound technique to measure angle of progression during labor. UOG.

[19] Dietz HP, Lanzarone V (2005). Measuring engagement of the fetal head: validity and reproducibility of a new ultrasound technique. UOG.

[20] Duke W, Shin M, Correa A, Alverson C (2010). Survey of knowledge, attitudes, and practice management patterns of Atlanta-area obstetricians regarding stillbirth. Womens Health Issues.

[21] Dupuis O, Ruimark S, Corinne D, Simone T, Andre D, Rene-Charles R (2005). Fetal head position during the second stage of labor: comparison of digital vaginal examination and transabdominal ultrasonographic examination. Eur J Obstet Gynecol Reprod Biol.

[22] Eggebø T, Hassan W, Salvesen K, Lindtjørn E, Lees C (2014). Sonographic prediction of vaginal delivery in prolonged labor: a two-center study. JUiO & Gynecology.

[23] Buchmann EJ, Libhaber E (2007). Accuracy of cervical assessment in the active phase of labour. BJOG.

[24] Barbera A, Pombar X, Perugino G, Lezotte D, Hobbins J (2009). A new method to assess fetal head descent in labor with transperineal ultrasound. Ultrasound Obstet Gynecol.

[25] Impey L, Hobson J, O'Herlihy C (2000). Graphic analysis of actively managed labor: prospective computation of labor progress in 500 consecutive nulliparous women in spontaneous labor at term. Am J Obstet Gynecol.

[26] Ghi T, Farina A, Pedrazzi A, Rizzo N, Pelusi G, Pilu G (2009). Diagnosis of station and rotation of the fetal head in the second stage of labor with intrapartum translabial ultrasound. UOG.

[27] Ghi T, Maroni E, Youssef A, Morselli-Labate AM, Paccapelo A, Montaguti E (2014). Sonographic pattern of fetal head descent: relationship with duration of active second stage of labor and occiput position at delivery. UOG.

[28] Gillor M, Vaisbuch E, Zaks S, Barak O, Hagay Z, Levy R (2017). Transperineal sonographic assessment of angle of progression as a predictor of successful vaginal delivery following induction of labor. UOG.

[29] Gunnarsson B, Skogvoll E, Jónsdóttir IH, Røislien J, Smárason AK (2017). On predicting time to completion for the first stage of spontaneous labor at term in multiparous women. BMC Pregnancy and Childbirth.

[30] Irani M, Kordi M, Lotfalizadeh M (2019). Methods of assessing the labor progress: A review study. Iran J Obstet Gynecol Infertil.

[31] Kameyama S, Sato A, Miura H, Kumagai J, Sato N, Shimizu D, Makino K, Terada Y (2016). Prediction of spontaneous vaginal delivery by transperineal ultrasound performed just after full cervical dilatation is determined. J Med Ultrason.

[32] Lavender T, Stine B (2020). Use of the partograph-current thinking. Best Pract Res Clin Obstet Gynaecol.

[33] Panchal V, Patel RD, Charel AK, Jikadara P, Mehta DA (2021). Prospective observational study of evaluation of progress of labour with partograph in primigravida and multigravida. Natl J Integr Res Med.

[34] Tabandeh A, Kashani E (2007). The prevalancy of cesarean among employed educated women of medical science groups in Gorgan. J. Gorgan Univ. Med. Sci.

[35] Khazardoost S, Ghotbizadeh Vahdani F, Latifi S, Borna S, Tahani M, Rezaei MA, Shafaat M (2016). Pre-induction translabial ultrasound measurements in predicting mode of delivery compared to bishop score: a cross-sectional study. BMC Pregnancy Childbirth.

[36] Bultez T, Quibel T, Bouhanna P, Popowski T, Resche-Rigon M, Rozenberg P (2016). Angle of fetal head progression measured using transperineal ultrasound as a predictive factor of vacuum extraction failure. UOG.

[37] Malvasi A, Montanari Vergallo G, Tinelli A, Marinelli E (2018). Can the intrapartum ultrasonography reduce the legal liability in distocic labor and delivery?. J Matern Fetal Neonatal Med.

[38] Malvasi A, Stark M, Ghi T, Farine D, Guido M, Tinelli A (2012). Intrapartum sonography for fetal head asynclitism and transverse position: sonographic signs and comparison of diagnostic performance between transvaginal and digital examination. J Matern-Fetal Neonatal Med.

[39] Popowski T, Porcher R, Fort J, Javoise S, Rozenberg P (2015). Influence of ultrasound determination of fetal head position on mode of delivery: a pragmatic randomized trial. ISUOG.

[40] Ghi T, Eggebø T, Lees C, Kalache K, Rozenberg P, Youssef A, Salomon LJ, Tutschek B (2018). ISUOG Practice Guidelines: intrapartum ultrasound UOG.

[41] Tutschek B, Braun T, Chantraine F, Henrich W (2011). A study of progress of labour using intrapartum translabial ultrasound, assessing head station, direction, and angle of descent. BJOG.

[42] Pereira S, Frick AP, Poon LC, Zamprakou A, Nicolaides KH (2014). Successful induction of labor: prediction by preinduction cervical length, angle of progression and cervical elastography. UOG.

[43] Yonetani N, Yamamoto R, Murata M, Nakajima E, Taguchi T, Ishii K, Mutauda N (2017). Prediction of time to delivery by trans-perineal ultrasound in second stage of labor. UOG.

[44] Ashley S, Helen C, Susan K, Colette M, Maggie S (2010). The purple line as a measure of labour progress: a longitudinal study. BMC Pregnancy Childbirth.

[45] Masturzo B, De Ruvo D, Gaglioti P, Todros T (2014). Ultrasound imaging in prolonged second stage of labor: does it reduce the operative delivery rate?. J Matern Fetal Neonatal Med.

[46] Barbera AF, Imani F, Becker T, Lezotte DC, Hobbins JC (2009). Anatomic relationship between the pubic symphysis and ischial spines and its clinical significance in the assessment of fetal head engagement and station during labor. ISUOG.

